# Undocumented UroLume Stent Presenting Years Later With Acute Urinary Retention and Infection in a Patient With Dementia

**DOI:** 10.7759/cureus.85351

**Published:** 2025-06-04

**Authors:** Omar Demachkie, Evan Ehsan, Andres Saucedo, Dulcinea D Jones, Sivarama K Kotikalapudi

**Affiliations:** 1 Medicine, William Carey University College of Osteopathic Medicine, Hattiesburg, USA; 2 Emergency Medicine, William Carey University College of Osteopathic Medicine, Hattiesburg, USA; 3 Orthopedic Surgery, William Carey University College of Osteopathic Medicine, Hattiesburg, USA; 4 Pediatric Medicine, William Carey University College of Osteopathic Medicine, Hattiesburg, USA; 5 Internal Medicine, Merit Health Wesley, Hattiesburg, USA

**Keywords:** artificial urethral sphincter, dementia, emergency cystoscopy, urinary retention, urolume stent

## Abstract

An 87-year-old male with dementia was admitted to the behavioral health unit for behavioral disturbances, including increased confusion, agitation - especially when taking medications - daytime somnolence with nighttime wakefulness, and disorientation. He rapidly deteriorated, becoming nonverbal and unresponsive to questions, due to urinary retention and infection caused by a retained UroLume stent placed approximately 30 years earlier. Despite stable vitals and a normal white blood cell count just days prior, his WBC acutely rose to 27,000 cells/mm^3^, prompting urgent urological intervention. The evaluation also revealed an undocumented artificial urinary sphincter (AUS), which further contributed to urinary retention and complicated management. This case emphasizes the importance of thorough history-taking, physical exams, and awareness of outdated implants, as the absence of documentation regarding both the UroLume stent and AUS in this 87-year-old patient significantly delayed diagnosis and treatment. It also highlights the need for meticulous clinical evaluation in elderly patients presenting with altered mental status.

## Introduction

Acute urinary retention (AUR) in elderly patients with dementia is often attributed to benign prostatic hyperplasia (BPH), medications such as anticholinergics and opioids [1], or neurological disorders like Parkinson’s disease, where dysfunction of the autonomic nervous system affects the normal control of bladder function [2]. Mechanical obstruction due to encrustation can occur with UroLume stents, as observed in this case. Urinary retention occurs postoperatively in 8-32% of patients who undergo artificial urinary sphincter (AUS) implantation, but it typically occurs early [3]. To the best of our knowledge, there have been no reports of this complication presenting so long after implantation. However, common causes of urinary obstruction, such as BPH and urolithiasis, are more frequently encountered in clinical practice. We present the case of an 87-year-old male whose symptoms, including increased confusion, agitation, daytime somnolence with nighttime wakefulness, and disorientation, were initially mistaken for dementia progression. However, upon further evaluation, including a bladder scan, imaging, and failed catheterization attempts, the true cause was revealed to be a retained UroLume stent and multiple bladder stones.

## Case presentation

An 87-year-old male with a history of coronary artery disease (CAD), hypertension (HTN), post-traumatic stress disorder (PTSD), and dementia on hospice was admitted to the behavioral health unit for worsening confusion, agitation, and restlessness. His dementia, which developed after a past brain hemorrhage and subsequent surgery, had progressively worsened, though he had been managed at home until recently. At home, he had become increasingly disoriented, exhibited sleep disturbances, and resisted taking medications.

**Figure 1:**
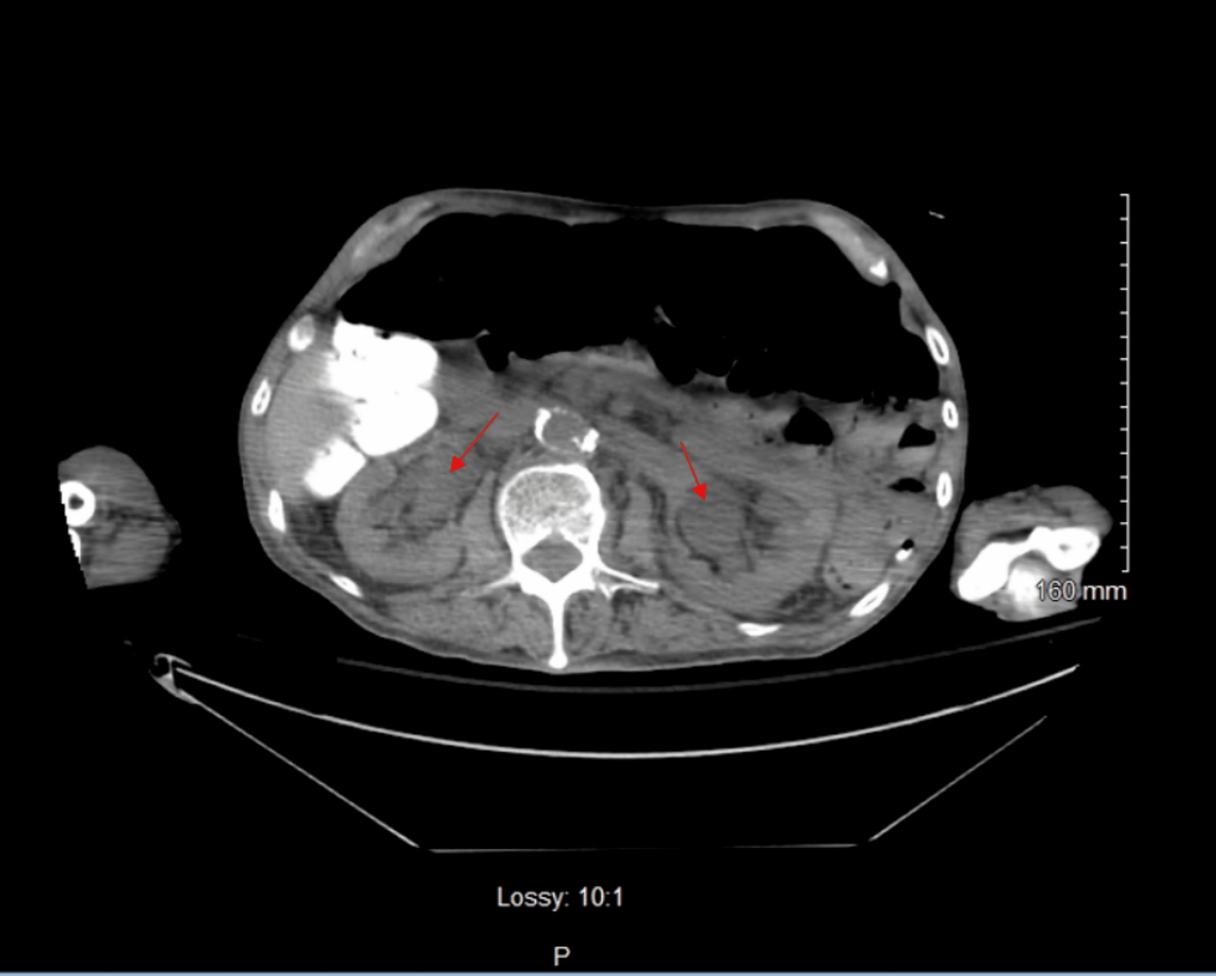
Axial non-contrast CT image at the renal level Mild-to-moderate bilateral hydroureteronephrosis, with red arrows pointing to both sides.

**Figure 2:**
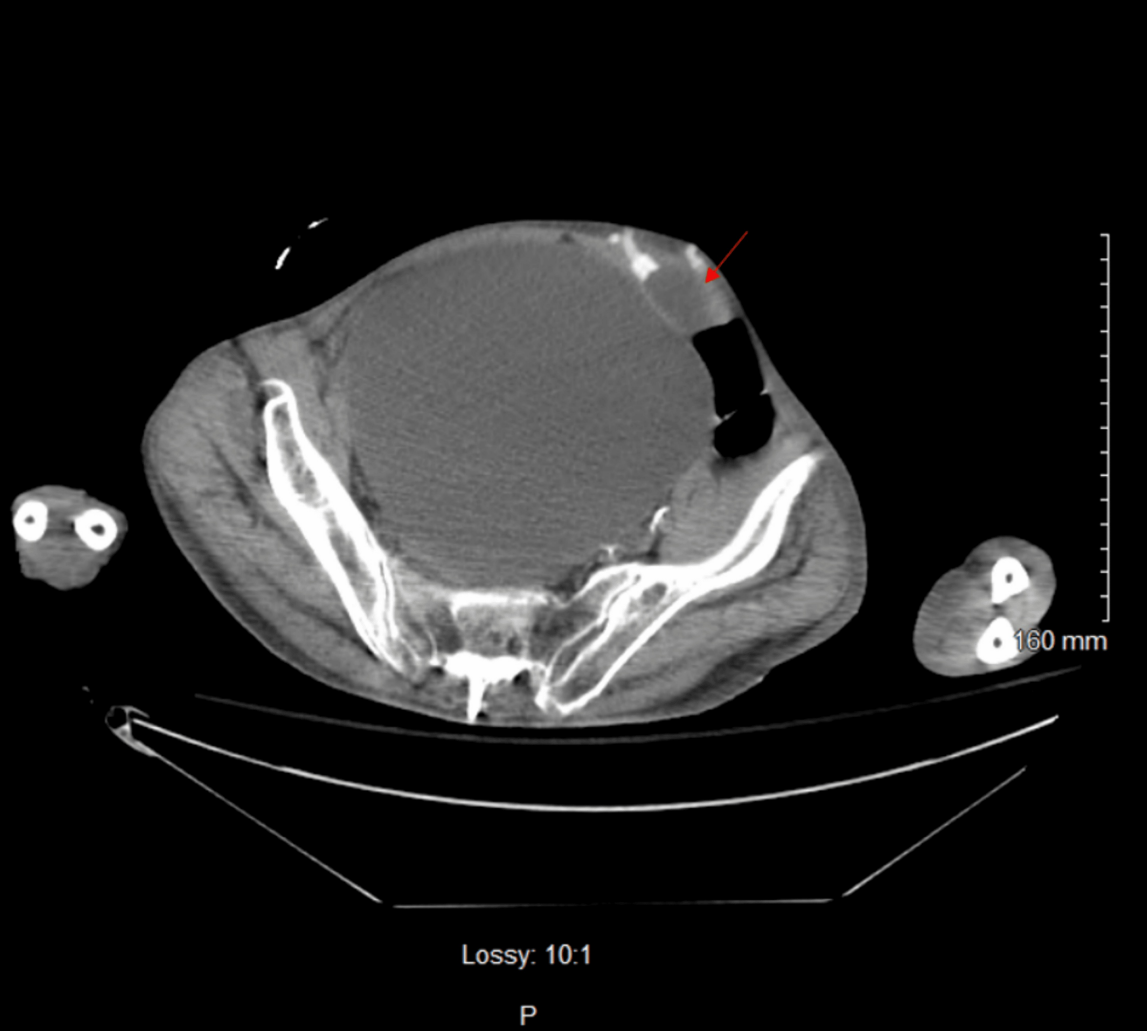
Axial non-contrast CT image at the bladder level The bladder is obstructed and distended, measuring 18 cm, with no apparent gross thickening of the bladder wall; a red arrow points to the abdominal balloon reservoir of the artificial urethral sphincter.

**Figure 3:**
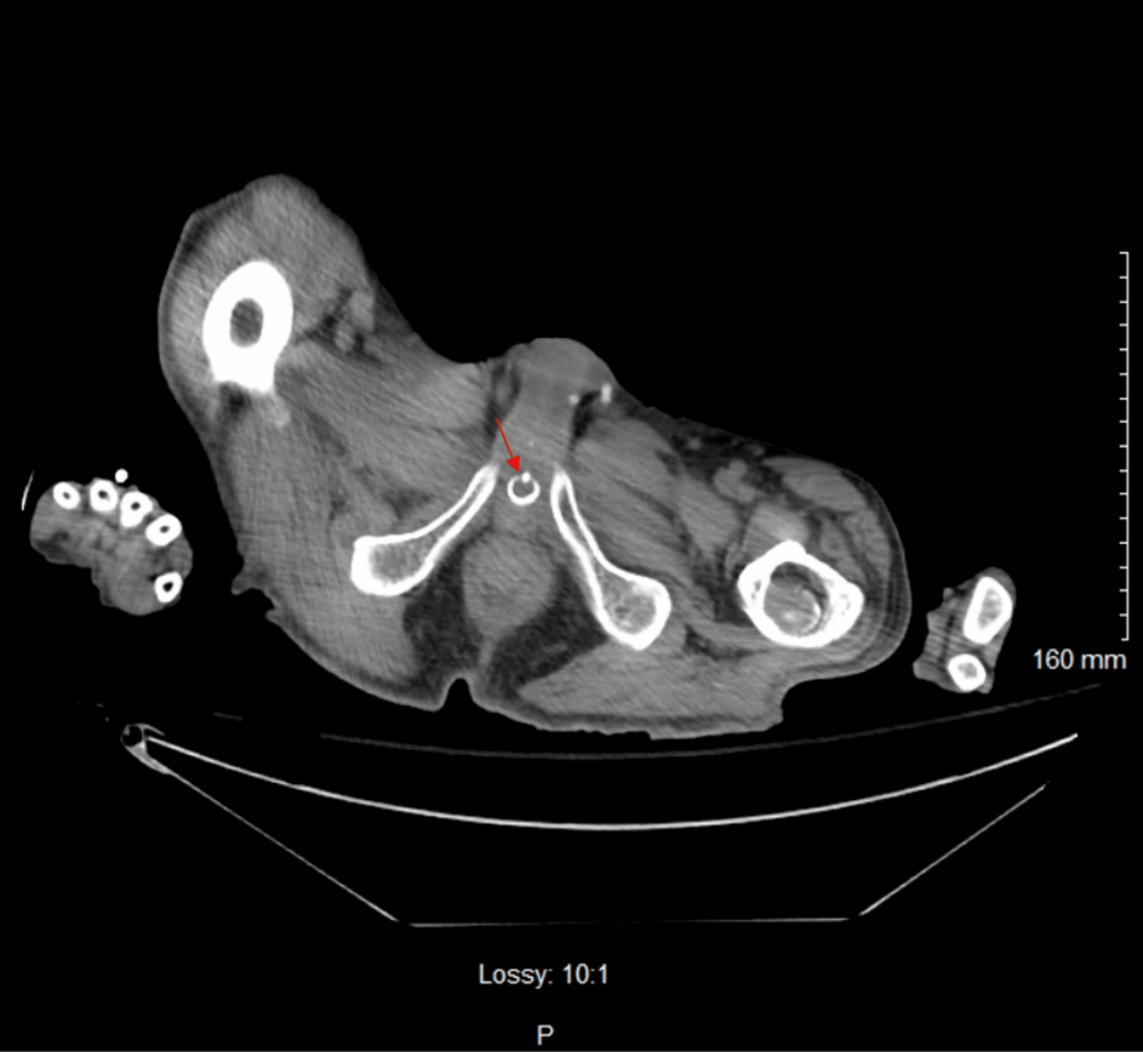
Axial non-contrast CT image at the prostate and penile region The prostate appears enlarged with multiple brachytherapy seeds and prior transurethral resection of the prostate (TURP). The red arrow points to the internal bladder-urethral sphincter.

Three days before admission, recent laboratory results, including a complete blood count (CBC), were unremarkable with a normal white blood cell (WBC) count of 6.7 × 10^3^ cells/mm^3^. Upon arrival, the patient was alert but combative, with a 3/6 holosystolic murmur and a firm, non-reducible mass in the left lower quadrant. The patient attributed the mass to a recent hernia repair surgery. However, within 48 hours of admission, his clinical picture deteriorated. He became minimally responsive to verbal commands and hypertensive (198/91 mmHg) and developed leukocytosis (Table 1). The differential diagnosis was expanded beyond psychological conditions. Urinalysis (UA) revealed red-colored, cloudy urine with a specific gravity of 1.010, proteinuria (30 mg/dL), trace leukocyte esterase, numerous red blood cells (too numerous to count (TNTC)), and few bacteria (Table 2). Physical examination at this point revealed suprapubic distension with a firm and spastic bladder. While these findings can suggest a urinary tract infection, in this case, they were more concerning for obstructive uropathy causing near-complete urinary retention and potential kidney injury rather than a primary infection. A bladder scan showed over 800 mL of retained urine. Attempts at Foley catheter placement with 14- and 16-French (Fr) catheters were unsuccessful. A computed tomography (CT) scan of the abdomen and pelvis revealed bilateral hydronephrosis (Figure 1), bladder distention (Figure 2), an enlarged prostate with multiple brachytherapy seeds, and a history of transurethral resection of the prostate (TURP), as well as an internal bladder-urethral sphincter (Figure 3), which was later determined to be an AUS by the urologist. Additionally, mild bilateral patchy basilar posterior airspace infiltrates consistent with pneumonia were noted.

**Table 1:** Complete blood count with differential and general chemistry H: high; L: low The percentage symbol (%) represents the relative proportion. The number symbol (#) refers to the absolute count.

Parameter	February 24, 2025	February 23, 2025	February 22, 2025	Reference range
Complete blood count				
White blood cells (WBC)	9.20	13.90 (H)	27.50 (H)	4.0-11.0 x 10^9/L
Red blood cells (RBC)	3.90	4.22	4.65	4.2-5.4 x 10^12/L
Hemoglobin (Hgb)	11.3 (L)	12.0	13.7	13.5-17.5 g/dL
Hematocrit (HCT)	35.2 (L)	38.1	41.6	40-50%
Mean corpuscular hemoglobin (MCH)	28.9	28.4	29.5	27-31 pg/cell
Mean corpuscular hemoglobin concentration (MCHC)	32.1 (L)	31.5 (L)	33.1	32-36 g/dL
Mean corpuscular volume (MCV)	90.1	90.1	89.4	80-100 fL
Platelet count	239	252	317	150-450 x 10^9/L
Mean platelet volume (MPV)	8.5	8.1	8.4	7.5-11.5 fL
Red cell distribution width (RDW)	14.1	13.9	13.6	11.5-14.5%
Differential				
Neutrophils (%) auto	76.2 (H)	88.4 (H)	96.2 (H)	40-70%
Lymphocytes (%) auto	14.0	5.5 (L)	1.1 (L)	20-40%
Monocytes (%) auto	7.5	5.4	2.4 (L)	2-8%
Basophils (%) auto	0.8	0.2	0.3	0-1%
Eosinophils (%) auto	1.5	0.5	0.0	0-5%
Neutrophils (#) auto	7.0	12.3 (H)	26.5 (H)	2.0-7.5 x 10^9/L
Lymphocytes (#) auto	1.3	0.8	0.3 (L)	1.0-3.0 x 10^9/L
Monocytes (#) auto	0.7	0.7	0.6	0.1-0.8 x 10^9/L
Basophils (#) auto	0.1	0.0	0.1	0.0-0.1 x 10^9/L
Eosinophils (#) auto	0.1	0.1	0.0	0.0-0.5 x 10^9/L
General chemistry				
Blood urea nitrogen	32	32	34	7-20 mg/dL
Creatinine	1.02	1.57	2.17	0.6-1.2 mg/dL
Estimated glomerular filtration rate (eGFR)	71	42	29	>60 mL/min/1.73 m²
Blood urea nitrogen (BUN)/creatinine ratio	31.4	20.4	15.7	10-20

**Table 2:** Urinalysis summary on February 22, 2025 UA: urinalysis; Ur: urine; WBC: white blood count; RBC: red blood cell; TNTC: too numerous to count

Parameter	Value	Normal range
UA macroscopic		
Ur color	Red	Yellow
Ur appearance	Cloudy	Clear to pale yellow
Ur specific gravity	1.010	≤1.005
Ur pH	6.5	5-7.5
Ur protein	30	Negative
Ur glucose	Negative	Negative
Ur ketone	Negative	Normal: negative
Ur nitrite	Negative	Normal: negative
Ur urobilinogen	0.2	Normal: 0.2
Ur leukocyte esterase	Trace	Negative
Ur collection source	Random	
UA microscopic		
Ur WBC	0-5	Negative
Ur RBC	TNTC	Negative
Ur bacteria	Few	Negative
Ur epithelial cells	Negative	Negative

**Figure 4:**
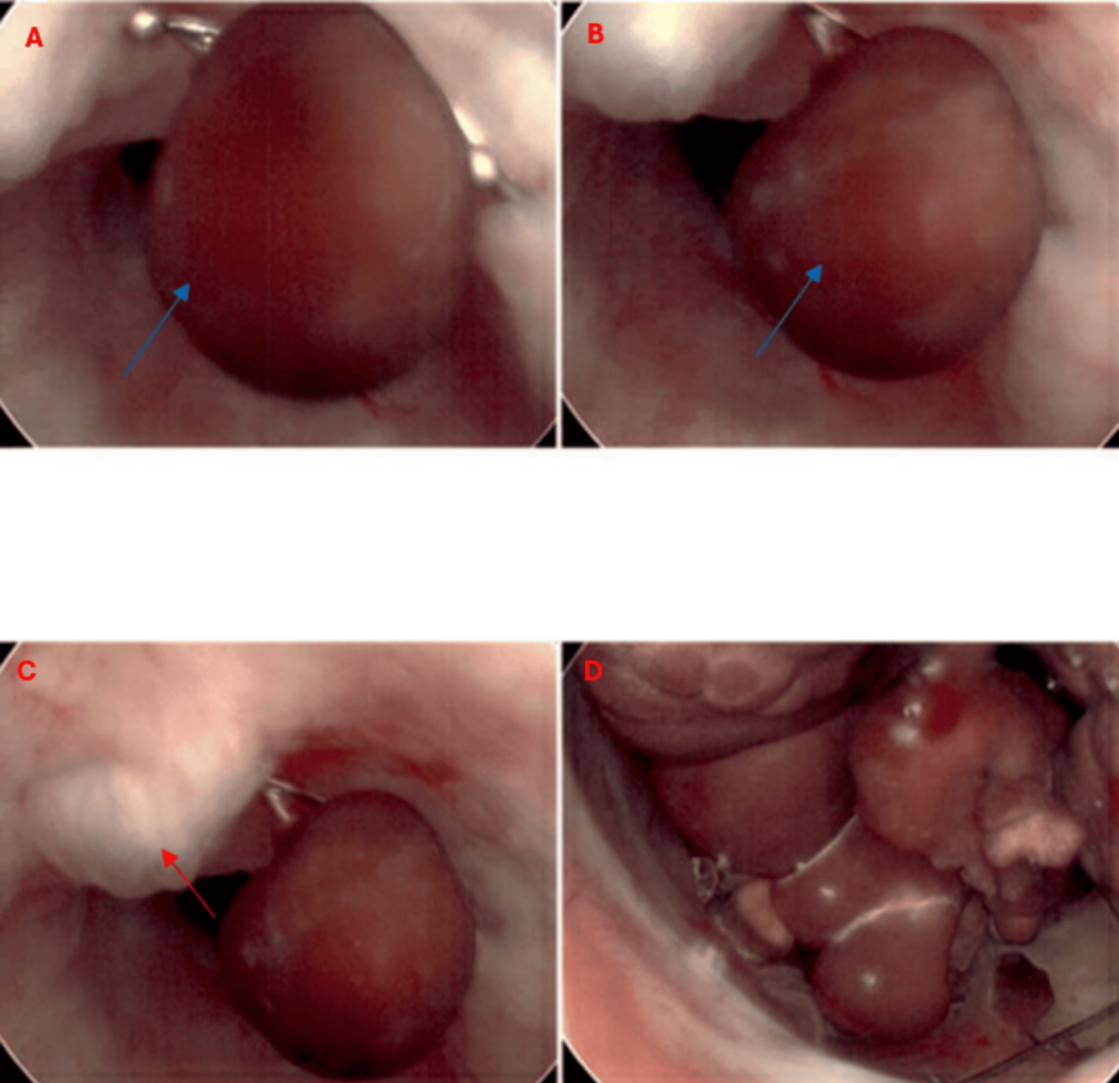
Cystoscopy Large bladder stones are seen in panels A and B (e.g., blue arrow), alongside an encrusted and heavily calcified UroLume stent in panel C (red arrow). Multiple large bladder stones are also visible in panel D.

A urologist was consulted after failed catheterization attempts and concern for urinary obstruction. During evaluation, an AUS was identified. Believing that previous failures may have resulted from an inflated AUS cuff, the urologist deflated the device and attempted catheterization with 10-, 12-, and 14-Fr catheters, but all attempts were unsuccessful. Given the persistent obstruction, a cystoscopy was performed, which disclosed significant obstruction of the prostatic urethra caused by a heavily encrusted UroLume stent that had been implanted decades earlier. The stent exhibited extensive encrustation, characterized by the accumulation of mineral deposits - likely calcium or other urinary salts - on its surface. This encrustation manifested as irregular, rough, and whitish deposits, giving the stent a markedly uneven and less smooth texture. The severe calcification of the stent ultimately impeded the advancement of the cystoscope into the bladder, preventing further visualization. Additionally, multiple large bladder stones were identified, further contributing to the obstruction (Figure 4). However, the stones could not be removed because they were corroded and fused to the bladder wall. Given the findings, a suprapubic catheter was placed using a trocar-assisted technique. The procedure was well tolerated, and the patient was transitioned to floor care for further management following cystoscopy and treatment of pneumonia.

The following day, the patient's blood pressure stabilized at 122/68, and his WBC count decreased to 13.9 (Table 1), likely due to the drainage from the catheter. He was also able to eat and communicate properly, which had been initially impaired. Despite not removing the stones, the patient's symptoms significantly improved, and his overall condition showed marked improvement, including the above findings as well as the absence of agitation, a calm demeanor, and a willingness to take his medication. Additionally, the urine culture collected post-catheterization showed no bacterial growth at two days.

## Discussion

This case emphasizes the importance of vigilant observation and thorough physical exams in elderly dementia patients. In patients with dementia, new or altered symptoms, such as confusion and agitation, can often be mistakenly attributed solely to the progression of cognitive decline. However, it is crucial to recognize that these symptoms may reflect underlying physical issues that are not immediately apparent, even despite unremarkable labs and vital signs. Dementia can mask acute illnesses, increasing the risk of misdiagnosis and delayed care. According to a report by the Agency for Healthcare Research and Quality (AHRQ), over 10% of both overdiagnosis and underdiagnosis occur in conditions like dementia, chronic obstructive pulmonary disease (COPD), Parkinson’s, heart failure, stroke, and myocardial infarction [4]. Additionally, 26% of hospitalized patients with pre-existing dementia lacked proper documentation of their diagnosis, potentially leading to mismanagement [5].

Urinary obstruction is one such condition that can be easily overlooked, particularly when initial UA findings are misinterpreted. It is absolutely possible to observe hematuria (red blood cells TNTC), proteinuria, trace leukocyte esterase, and cloudy, red urine in the setting of obstruction without active infection. Obstruction, especially prolonged, can lead to bladder wall irritation, pressure-induced renal tubular damage, or even microhemorrhage, all of which can mimic or overlap with typical infectious UA patterns. These findings must be carefully correlated with clinical context, particularly in patients unable to articulate symptoms, such as those with dementia.

Despite initially stable vitals and a normal white blood count, the patient rapidly developed AUR, severe leukocytosis, and HTN. The presence of normal labs and stable vital signs - paired with the patient’s cognitive decline - initially led to the misattribution of his symptoms to dementia progression. However, this abrupt clinical deterioration highlights the importance of frequent reassessment rather than relying solely on initial laboratory results. Urethral obstruction can progress quickly, leading to life-threatening complications if not promptly diagnosed.

This case is particularly notable due to the presence of a UroLume stent, a once-common implant for bladder outlet obstruction that was largely abandoned due to complications like encrustation and obstruction. Developed in the 1980s, the UroLume is a permanent, self-expanding metallic stent designed to maintain urethral patency in patients with urinary obstruction. Once deployed, it expands to approximately 30 Fr in diameter and is delivered using a 24 Fr system for ease of placement during endoscopic procedures [6]. Initially recommended for men who were not candidates for major surgery but still required urethral patency, it was also used in cases of radiation-induced strictures [7]. However, studies reported high complication rates, including hematuria, chronic pain, infections, and stone formation, often necessitating removal. One study found a 35% complication rate, including gross hematuria and intermittent clot retention, in patients who received the UroLume stent for BPH [8]. Another study linked permanent urethral stents, such as the UroLume, to encrustation, urinary tract infections, chronic pain, and stone formation, reinforcing the need for their removal [9].

The presence of a long-residing UroLume stent, undocumented in the patient’s recent medical records, suggests that the delayed complications may have arisen in part due to patient-related factors such as poor compliance with follow-up care and memory lapses over the years. These factors likely contributed to the progression of urinary obstruction and the development of associated clinical symptoms in advanced age.

The calcified UroLume stent was left in place due to severe calcifications and adhesions, making its removal risky and potentially harmful, especially considering the patient's advanced age and complex medical history. Similarly, the stones were not removed, as their extraction was deemed too difficult and dangerous, with the risks outweighing the potential benefits. After discussing the patient's condition and options with the family, including the impact on quality of life with or without surgery, the decision was made to insert a suprapubic catheter for symptom management. The patient was then transferred to home hospice care to focus on comfort and quality of life.

A similar case report describes a 24-year-old male who developed malignant HTN and azotemia secondary to bladder outlet obstruction, with significant improvement following transurethral dilation [10]. While the patient in this case report initially did not have azotemia on admission, this may be explained by either the presence of intermittent or partial bladder outlet obstruction or by the patient’s low muscle mass, as was the case here. For example, serum creatinine is influenced by muscle mass; individuals with low muscle mass may produce less creatinine, resulting in deceptively normal values even in the presence of reduced glomerular filtration rate [11]. Therefore, a normal creatinine level does not necessarily exclude kidney dysfunction.

The calcified UroLume stent was left in place due to severe calcifications and adhesions, making its removal risky and potentially harmful, especially considering the patient's advanced age and complex medical history. Similarly, the stones were not removed, as their extraction was deemed too difficult and dangerous, with the risks outweighing the potential benefits. After discussing the patient's condition and options with the family, including the impact on quality of life with or without surgery, the decision was made to insert a suprapubic catheter for symptom management. The patient was then transferred to home hospice care to focus on comfort and quality of life.

According to an article by National Health Service England, managing an AUS properly during catheterization is essential to avoid complications. The guidelines stress that the AUS cuff must always be deflated before catheter insertion to prevent tissue damage, trauma, or infection. If this step is not performed, serious harm can occur [12]. Identifying an AUS during a physical exam can be difficult because of its internal placement. Typically, the device includes a cuff around the urethra, a control pump in the scrotum, and a pressure-regulating balloon in the abdomen. Palpation of the scrotum may reveal the pump, which can aid in recognizing the presence of an AUS. However, imaging studies such as CT or MRI are more effective in visualizing the entire device and assessing its condition [13]. The study by Capra and Chou (2018) on the cross-sectional appearance of common implants revealed the following features: 1) the urethral cuff appears as a circumferential structure surrounding the urethra; 2) the scrotal pump is visible within the scrotum, identifiable by its characteristic shape and location; and 3) the abdominal balloon reservoir is located in the abdomen, typically near the bladder, and is distinguishable based on its size and configuration.

The current recommendation from the National Institute of Diabetes and Digestive and Kidney Diseases (NIDDK) for obtaining a CT scan in cases of urinary retention is when symptoms remain unresolved or are severe, particularly if initial evaluations (e.g., bladder scan or ultrasound) do not clarify the cause. A CT scan is also recommended if the physician suspects that the retention is due to an obstruction or in cases involving sepsis, hematuria, or recurrent urinary tract infections [14].

​AUR is a urologic emergency that requires immediate intervention. This intervention should occur as soon as retention is clinically suspected or confirmed via ultrasound [15]. For patients with a history of urethral stricture, injury, bladder or urethral surgery, or pelvic/perineal trauma that may have altered urethral anatomy, it is advisable to urgently refer them for urologic evaluation rather than attempting standard catheterization. These individuals may require alternative approaches, such as using a coudé-tip catheter, endoscopic catheter placement via cystoscopy, or suprapubic catheterization, to ensure safe and effective management [16].

The decision to escalate care to cystoscopy and suprapubic catheterization was critical in preventing further complications such as renal failure or septic shock. A study suggests that integrating bedside retrograde urethrogram and retrograde urethroscopy can help identify urethral pathology, allowing for dilatation and per urethral catheterization, thus avoiding suprapubic catheter placement [17].

## Conclusions

This case reinforces the importance of maintaining a high index of suspicion for urinary obstruction in elderly patients with altered mental status. Clinicians should always consider past urological interventions, even if undocumented, and act promptly to prevent severe complications. Ultimately, early urological intervention, including cystoscopy and suprapubic catheter placement, played a crucial role in stabilizing this patient and preventing further complications.
